# How do patients with high cardiovascular risk evaluate online health information? A qualitative study

**DOI:** 10.1186/s12875-023-02182-7

**Published:** 2023-11-15

**Authors:** Hooi Min Lim, Chirk Jenn Ng, Adina Abdullah, Adam G. Dunn

**Affiliations:** 1 Department of Primary Care Medicine, Faculty of Medicine, Universiti Malaya, Kuala Lumpur, Malaysia; 2 Department of Research, SingHealth Polyclinics, 167 Jalan Bukit Merah Connection One (Tower 5)#15-10, Singapore, 150167 Singapore; 3 Duke-NUS Medical School, Singapore, Singapore; 4 Biomedical Informatics and Digital Health, School of Medical Sciences, Faculty of Medicine and Health, The University of Sydney, Sydney, NSW Australia

**Keywords:** Consumer health informatics, Health information, eHealth literacy, Misinformation, eHealth, Internet, Trust, Primary care

## Abstract

**Background:**

People are exposed to variable health information from the Internet, potentially influencing their health decision-making and behaviour. It remains a challenge for people to discern between good- and poor-quality online health information (OHI). This study explored how patients evaluate and determine trust in statin-related OHI in patients with high cardiovascular risk.

**Methods:**

This qualitative study used vignettes and think-aloud methods. We recruited patients from a primary care clinic who were at least 18 years old, had high cardiovascular risk and had previously sought OHI. Participants were given two statin-related vignettes: Vignette 1 (low-quality information) and Vignette 2 (high-quality information). Participants voiced their thoughts aloud when reading the vignettes and determined the trust level for each vignette using a 5-point Likert scale. This was followed by a semi-structured interview which was audio-recorded and transcribed verbatim. The transcripts were coded and analysed using thematic analysis.

**Results:**

A total of 20 participants were recruited, with age ranging from 38–74 years. Among all the high cardiovascular-risk participants, eight had pre-existing cardiovascular diseases. For Vignette 1 (low-quality information), five participants trusted it while nine participants were unsure of their trust. 17 participants (85%) trusted Vignette 2 (high-quality information). Five themes emerged from the analysis of how patients evaluated OHI: (1) logical content, (2) neutral stance and tone of OHI content, (3) credibility of the information source, (4) consistent with prior knowledge and experience, and (5) corroboration with information from other sources.

**Conclusion:**

Patients with high cardiovascular risks focused on the content, source credibility and information consistency when evaluating and determining their trust in statin-related OHI. Doctors should adopt a more personalised approach when discussing statin-related online misinformation with patients by considering their prior knowledge, beliefs and experience of statin use.

**Supplementary Information:**

The online version contains supplementary material available at 10.1186/s12875-023-02182-7.

## Background

The Internet has become the main source of health information worldwide due to its high accessibility. Over 70% of people from developed countries seek health information from the Internet [1]. In low-middle-income countries (LMICs), the prevalence of online health information-seeking is increasing, reported 50–70%, with the increase in smartphone usage [2, 3]. People seek online health information (OHI) to support their health decision-making, change in health behaviour, and health service utilisation [4, 5]. Better-informed patients with knowledge from online information were more engaged in their health decision-making [6, 7]. However, low-quality information or misinformation exposure might negatively affect patients’ behaviour [8]. For example, the influence of misinformation about vaccines is observed in social media, where people exposed to negative information are more likely to have negative opinions [9].

While internet-based information is helpful for patient education and health information dissemination [10], not all online health resources deliver high-quality health information. For example, a study evaluating the quality of 400 websites related to common cancers showed that online cancer information was highly variable, with a lack of accountability features, such as disclosure of authorship and source attribution, and poor readability [11]. Similarly, a systematic review showed that online websites about preoperative fasting provided inaccurate and out-of-date recommendations to patients [12]. With the highly variable quality of OHI, people face challenges when evaluating its credibility, accuracy and reliability [13].

eHealth literacy is the degree to which people access, understand, evaluate, and use OHI to make appropriate health decisions [14]. A systematic review shows that low eHealth literacy is associated with a lower ability to evaluate the quality of OHI [15], which includes assessment of the credibility, relevance, understandability, accuracy, readability and applicability of the information [16]. In the process of evaluating OHI, people decide whether or not to trust the information based on their perceived quality of the information [17]. Trust in information is a psychological state which indicates a positive belief about the perceived reliability, dependability, and confidence in the words, oral or written statements of another individual or group [18]. Trust in information has been used as a measure in health information behaviour research; it is associated with readiness and intention to use the information for making health decisions [19].

Few studies examine how people evaluate OHI and decide whether or not to trust OHI. A review reported that design features (i.e. layout of the website, interactive features and presence of contact details) and content features (i.e. content objectivity, readability and familiarity) are the main factors that influence trust in information [18]. Some studies were conducted among students or healthy people; however, the findings may not be generalisable to patients with the health conditions [20, 21]. Most research on trust and credibility of OHI was conducted in the United States and the United Kingdom [18]; there is a lack of data from LMICs, whose populations have a lower health literacy and poorer ability to evaluate OHI [13, 15, 22]. Further research is needed to understand how patients evaluate and trust online information in LMICs.

In this study, we seek to understand how patients with high cardiovascular risks evaluate and trust OHI in the context of statin use. Although the benefits of statins outweigh the risks of side effects [23], studies have found that some patients with high cardiovascular risks often do not adhere to statins because of perceived lack of efficacy and fear of side effects [24]. One of the reasons for this is that statin-related health information in social media tends to emphasise the side effects and challenge the research evidence on statin efficacy [25, 26]. This may potentially affect patients’ perception of statins and how they decide on statin initiation and adherence [27, 28]. Using statin-related OHI as an example, we aimed to explore how patients with high cardiovascular risk evaluate OHI when making a decision on statin use.

## Methods

### Study design

The reporting of this study followed the Consolidation Criteria for Reporting Qualitative Studies (COREQ) [29]. This qualitative study used an interpretive description approach to inductively analyse how people evaluate OHI and determine their trust. In this study, we used a vignette technique to elicit rich and detailed views from participants because they may find it difficult to recall the experience of how they evaluated OHI [30]. We provided two statin-related vignettes to the participants. Vignettes acted as a clue to trigger participants to speak about how they evaluate the OHI, allowing participants to discuss the factors that affect their trust in OHI without forgetting the relevant content [31].

### Study setting and participants

The study was conducted at a primary care clinic which is part of a university teaching hospital in Kuala Lumpur from June to October 2021. This primary care clinic is located in an urban area and is attended by 300–400 patients daily. Patients attending the clinic were from different ethnic backgrounds, i.e. Malay, Chinese and Indian, and 50–60% were older than 60.

We selected patients with a high cardiovascular risk because statin-related OHI would be relevant to them and could potentially influence their decision on statin use. The inclusion criteria for this study were: age ≥ 18 years; had a high cardiovascular risk where statin use was recommended as per guideline (pre-existing cardiovascular disease, diabetes mellitus, chronic kidney disease ≥ stage 3, or Framingham General Cardiovascular Disease risk score > 20%) [32]; and had sought or encountered OHI. Patients unable to read English, Malay, or Chinese were excluded from this study.

### Vignettes

We chose vignettes that target people currently taking statins or for whom statins were recommended. The vignettes were available in three languages: English, Malay and Chinese; this allowed the patients to select the vignette in the language they were proficient in. We searched the vignettes from online health websites and social network platforms which were reported as popular sources of OHI in our previous study conducted at the same setting [2]. We also searched for information on websites from both local and international websites such as websites from the United Kingdom, the United States of America, China and Singapore, which were known to be commonly accessed by people in Malaysia [2].

Vignette 1 uses low-quality information about statins shared on social media while Vignette 2 uses high-quality, evidence-based information from an accredited organisation (Table 1). We determined the quality of vignettes using the DISCERN tool (range of score 15–75) [33]. The DISCERN scores for Vignette 1 (low-quality information) and Vignette 2 (high-quality information) were 31–32 and 63–71 respectively (see Additional file 1).

**Table 1:** Summary of vignettes

**Vignette 1: poor-quality information**
**Source:** Vignette 1 was a post from a Facebook channel using a name similar to the patient education website of the Ministry of Health; however, this Facebook channel was not endorsed by the Malaysian Ministry of Health
**Content:** This vignette stated that statins bring high profits to the pharmaceutical industry
It highlighted statin side effects: increased diabetes risk, liver failure, kidney failure, cataracts, muscle weakness and inflammation. It questioned the need for statins to prevent cardiovascular diseases because cholesterol is not the leading cause
**Source attribution:** This vignette cited a few well-established journals and institutions, such as the American Journal of Cardiovascular Drugs and the ‘Harvard’ study. It also quoted sentences from doctors
**Writing style and tone:** This vignette was written in a non-professional manner with a threatening tone e.g. *“In Malaysia, if you have been given a statin drug prescription by doctors, then be prepared for your final days.”*
**Vignette 2: High-quality information**
**Source:** This vignette was obtained from the official healthcare institutional website from the United States, with the title “Statin side-effects: Weigh the benefits and risks.”
**Content:** This vignette stated the indication of statins and listed the statin side-side effects: muscle pain and damage, liver damage, increased blood sugar or type 2 diabetes and neurological side effects. It explained weighing the benefits of statins against the risk of side effects and the rarity of the severe side effects. Other content included who is at risk of statin side effects, drugs and food interaction with statins, and how to relieve statin side effects. It advised patients to seek doctors’ advice if they have any concerns or experience any side effects
**Source attribution**: This vignette has a reference section citing 11 published articles
**Writing style and tone:** This vignette was written in a professional manner with a neutral tone in advising patients about statins

### Sampling and recruitment

We used a purposive sampling method to achieve maximum variation; we recruited information-rich cases based on their age, ethnicity, educational level and statin use for primary or secondary prevention. We identified participants who met the inclusion criteria based on their electronic medical records. We purposively selected participants based on their sociodemographic characteristics. Researchers approached potential participants during their clinic visits. A researcher (HML) asked the participants whether they had previous experience of seeking or encountering health information related to statins on the internet. All the participants were approached and recruited by the same researcher (HML). A participant information sheet was used to explain the objectives and methods of this study. Participants were given sufficient time to read the study information, asked questions before deciding whether or not to participate in the study. A written consent was taken for those who agreed to participate.

### Data collection

**Fig. 1:**
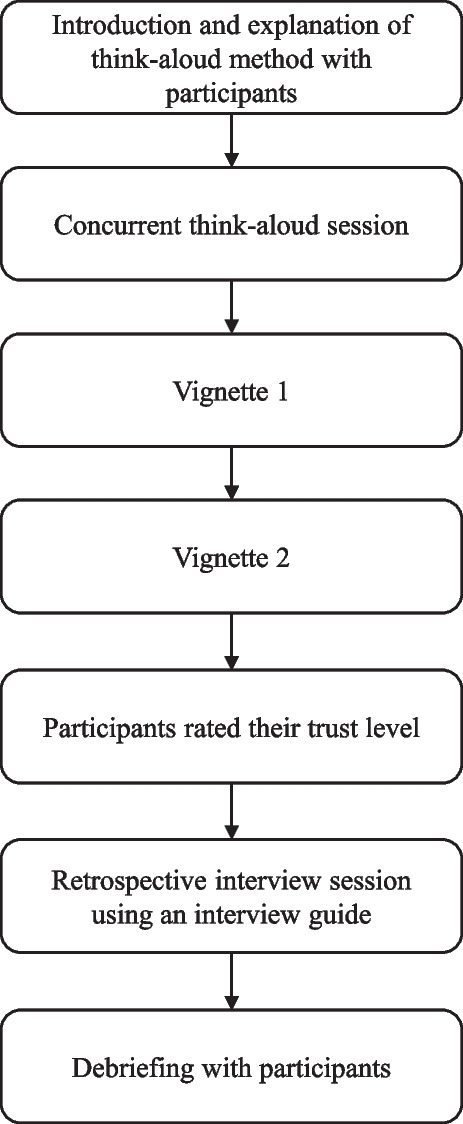
Flowchart of data collection

We used a think-aloud method, where participants think aloud their thoughts, allowing us to understand participants thinking when they read through the vignettes [34]. Participants were given a task to decide whether they would trust the vignettes while reading them from a tablet. There were two sessions in the interview: a concurrent think-aloud session and a retrospective interview session (Fig. 1). For the concurrent think-aloud session, participants were asked to think aloud while reading the vignettes. After the concurrent think-aloud session, participants were asked to rate the trust of each vignette using a 5-point Likert scale (1, No trust at all – 5, Mostly trust it). For the subsequent retrospective interview session, we asked the participants to elaborate and explain what has been verbalised in the concurrent think-aloud session. We also asked participants how they evaluate and rate the trust level for each vignette. For the retrospective interview session, we used a semi-structured interview guide (see Additional file 2), developed based on the models of trust in internet-based health information by Harris et al. [19] and Sillence et al. [35], literature review and discussion with the research team. The interview guide was revised after pilot-testing the interview with four participants.

Individual interviews were conducted; most were performed face-to-face in a quiet consultation room, while a few participants were interviewed virtually via the Zoom platform due to personal preference. For virtual interviews, the vignettes were shown using the Zoom shared screen function. The interviews were carried out by a researcher (HML), who was trained in qualitative interviewing and proficient in English, Chinese, and Bahasa Malaysia. The interviews were conducted in a language preferred by the participant. HML is a family physician practising in the same clinic setting, which has 30 family physicians and trainees. To avoid conflict of interest and the possibility of coercion, none of her patients were recruited into the study. HML introduced herself as a PhD student and a researcher (rather than a doctor) during the interviews to encourage patients to express their actual thoughts and feelings, and to avoid socially desirable responses. After the interview, participants had a debriefing session with the researcher (HML). The researcher discussed each vignette with the participant, allowed them to ask questions and provided the correct health information to them. This study obtained ethical approval from the Medical Research Ethics Committee (MREC), University of Malaya Medical Centre (MREC ID No.: 2021324–9981).

### Data analysis

All recorded interviews were transcribed verbatim by two professional transcribers. A researcher (HML) checked all the transcripts for accuracy. We used a thematic analysis method to code, analyse and report the data [36]. Two researchers (HML and CJN) familiarised themselves with the first two transcripts and generated the initial codes independently. Both researchers compared and discussed the codes and agreed on a coding framework, which guided the subsequent coding of the rest of the transcripts. HML coded the remaining transcripts. Both researchers met regularly throughout the coding process to discuss new codes that emerged and reach a consensus on all coding. Subsequently, both researchers sorted and categorised the codes into themes. We reviewed the themes by checking the coherence of the data within each theme. Any disagreement of the codes and themes between HML and CJN were resolved through consensus or discussion with the other members of the research team (AGD and AA). Concurrent data collection and analysis were performed iteratively to identify areas for exploration in the subsequent interview and to determine whether the data was saturated. Data saturation was reached after 20 interviews where no new themes emerged from data analysis and field notes. The data were analysed using Atlas.ti version 8 software.

## Results

A total of 20 patients were recruited and interviewed in this study (Table 2). The age of the patients ranged from 38 to 74 years. All participants had a high cardiovascular risk; eight had pre-existing cardiovascular diseases. Out of 20 participants, two had stopped taking statins for many years while one had refused to start statins. For Vignette 1 (low-quality information), five participants trusted it while nine participants were unsure of their trust. 17 participants (85%) trusted the Vignette 2 (high-quality information). There was no similar sociodemographic characteristics identified in the 5 participants who trusted Vignette 1.

**Table 2:** Characteristics of participants

Characteristics	N (%)
Age (years)	
30–39	2 (10)
40–49	2 (10)
50–59	5 (25)
0–69	8 (40)
> 70	3 (15)
Gender	
Male	11 (55)
Female	9 (45)
Ethnicity	
Malay	8 (40)
Chinese	8 (40)
Indian	4 (20)
Primary language	
English	11 (55)
Bahasa Malaysia	6 (30)
Chinese	3 (15)
Educational level	
Secondary school	5 (25)
Diploma	5 (25)
Degree	5 (25)
Master/PhD	5 (25)
Trust in Vignette 1(low-quality information)	
No trust at all	0 (0)
No trust	6 (30)
Not sure	9 (45)
Trust it	5 (25)
Mostly trust it	0 (0)
Trust in Vignette 2(high-quality information)	
No trust at all	0 (0)
No trust	1 (5)
Not sure	2 (10)
Trust it	14 (70)
Mostly trust it	3 (15)
Current use of statins	
Yes	17 (75)
No	3 (15)

Five themes emerged from the analysis of how patients evaluated OHI: (1) Logical content, (2) Neutral stance and tone of OHI content, (3) Credibility of the information source, (4) Consistent with prior knowledge and experiences, and (5) Corroboration with information from other sources.

### Theme 1: Logical content

Most participants focused on the content when evaluating the credibility of the vignettes. Some participants explained that they assessed the OHI based on their ‘common sense’ and judgement. Participants did not trust the vignette which contained information that did not sound ‘logical’.

*“I think some of, what is being mentioned here is actually have to do with common sense…because I tend to use my common sense, I make my own judgement you know, I feel...” (P2, Vignette 2)*

*“That was something that’s logical. Okay then, that I’ll accept it.” (P1)*

Upon reading Vignette 1, which mentioned diabetes as one of the statin side effects, some participants expressed their disagreement with the content as they believed doctors would not prescribe a medication that caused harm.

*“I don’t trust, it doesn’t sound logical, not logical. Because the doctor will not give us medication that will harm us, right?” (P8, Vignette 1)*

*“It (statin) is approved by Malaysia, all hospitals are giving statins…so I don’t think it’s a genuine info. (P15, Vignette 1)*

### Theme 2: Neutral stance and tone of OHI content

Participants trusted OHI with a balanced content, stating both benefits and risks of statins.

*“It looks convincing to me, and quite neutral, whether a person should take statins or should not take statins. Quite neutral and not biased to promote people taking statins or to frighten not to take statins. After I read this article, I can trust this article more.” (P16, Vignette 2)*

Participants distrusted OHI which was biased and skewed towards the risks of statins. Some participants perceived that Vignette 1 was trying to condemn the pharmaceutical company.

*“It looks to me as though like you know, the writer is trying to… like to…. How do I say? Condemn the company.” (P5, Vignette 1)*

*“I saw the information just now and it looks like it wants to ban someone. I think it is being too extreme.” (P4, Vignette 1)*

They trusted the OHI which was written in a professional manner with a neutral tone. Participants distrusted OHI which sounded threatening by overstating the risks of statins.

*“They’ve talked too much on the risks, Okay, and they overstated…but I don’t think that would be that severe.” (P1, Vignette 1)*

A participant expressed distrust in Vignette 2 (high-quality information), which she perceived as over-persuasive. She disliked the way the author tried to convince people to take statins after mentioning the potential side effects of statins.

*“This whole article seems like very bent on making you to take statins drug because it kinds of tell you it’s very safe… I will find it a bit annoying that it tries so hard to convince me that it seems obvious that there’s a lot of side effects.” (P6, Vignette 2)*

A participant expressed her trust in Vignette 2 because it recommended patients consult a doctor if they have any doubt about statins after reading the information.

*“It was quite consoling. Even though they have given the information, they talk about side effects, they are not hiding it…but they give you an option - not to be scared but consult your doctor.” (P12, Vignette 2)*

### Theme 3: Credibility of the information source

Most participants trusted OHI written or endorsed by credible and reputable healthcare organisations such as government institutions, universities and hospitals. Participants expressed their trust in the research conducted by those institutions.

*“Ya, if it comes from a good source, from a reputable source like a university, you know they have done a lot of research, Centre of Disease Control CDC in Geogia, or the Oxford Medical School you know…from Mayo Clinic, that is a well-known hospital in the world” (P3, Vignette 2)*

*“Okay, this article is more credible because it mentions FDA (U.S. Food and Drug Administration), so okay, can believe a bit more.” (P2, Vignette 2)*

However, some participants trusted the low-quality information in Vignette 1 because it named reputable organisations in the content. Although the participants were aware of the biased content in Vignette 1, they hesitated when Vignette 1 cited the research from a reputable university.

*“ It just put Harvard study, Oxford study, Cambridge study, all these very established universities… of course you will be quite impressed with their studies, right?” (P11, Vignette 1)*

Participants preferred and trusted information if they were written by medical professionals; this was illustrated in Vignette 1, where low-quality OHI was trusted by participants. When the author was not clearly stated in the vignettes, participants tried to guess the background and qualification of the author based on the content and writing style.

*“It seems it has been written by somebody who knows a lot of pharmaceutical stuff like statins... And also, most likely, the author must have at least a medical degree as well, comments on the effect of statins on various functions of the body like muscle damage and so on.” (P16, Vignette 1)*

*“This information is written by a doctor, I trust, I trust.” (P7, Vignette 1)*

Some participants trusted the information shared by others who experienced similar health conditions. They perceived that the experiences shared by someone like them would be genuine.

*“Some were just individuals who had experienced it personally… You’ve found that more impactful, that means that person has gone through it themselves, but of course, you will never know whether that’s real or they are just putting it out there, but the question you would ask yourself is why would anybody simply just put it out, what possible benefit could they have, maybe they just want to share with others to know better, you know?” (P6)*

### Theme 4: Consistent with prior knowledge and experiences

Most participants trusted OHI that concurred with their personal knowledge and experiences. They had more trust in OHI when the content was consistent with the information they had previously encountered.

*“According to the information that I read…those books, what it said compared to yours (vignette) are… are very similar, so I trust this a lot, this one…” (P10, Vignette 2)*

*“I suppose this is trustworthy because this is the type of knowledge that I have before.” (P11, Vignette 2)*

A participant expressed that he trusted the OHI in Vignette 1 (poor-quality information) because he had read similar information about statins bringing profits to pharmaceutical companies.

*“You read anywhere, they said statins enrich the big pharma… because any medicine, it’s a lot of money…I can see every patient they give simvastatin. You see…my wife is taking, my mother is taking… (P9, Vignette 1)*

They trusted the vignette when the information was consistent with situations or side effects they had experienced.

*“Okay, this article is more credible and I do follow statin, take your break from statin therapy. I revert to that triglyceride medication, switch to another statin drug which happened to me also, change your dose, dose which also happened to me.” (P2, Vignette 2)*

*“Ha! but now I know that this maybe… my sugar level cannot come down because of the statins, because they said that statins can cause blood sugar type 2 to go up, so as a doctor who prescribes the medicine, they should look at it.” (P9 - after reading Vignette 2 about statins causing diabetes)*

Conversely, they had less trust in the OHI when they did not experience any of the statin side effects as mentioned in the vignettes.

*“The one (Vignette 1) with 3 marks. It says that it (statin) has a lot… a lot of side effects…I took it (statin) but did not have any side effects.” (P18, Vignette 1)*

Participants trusted the OHI in Vignette 1 (poor-quality information) because the content was consistent with their prior perceptions of statins. They perceived that statins were ‘poisonous’ and would damage their liver and kidneys. The negative views about statins were consistent with the content of Vignette 1, which emphasised the side effects of statins.

*“In my mind, I always thought this drug (statin) is bad for me…I have this preconceived notion, I just think it (statin) is not good… In addition, your report (Vignette 1) is in line with my view, so I believe it.” (P10, Vignette 1)*

### Theme 5: Corroboration with information from other sources

Most participants expressed that they would check the consistency of information from multiple sources to corroborate the information they encountered.

*“…for internet information, I wouldn’t just accept it, unless I came across it, very frequently on medical website, medical journals… I look for those similarities.” (P1)*

*“Aa like this I will compare…so this website said cholesterol is good and then I have to go… to another one to confirm, the third one said good then I accept it. If this one said good, this one said not good then I reject it immediately.” (P17)*

A participant revealed that she would stop taking statins if she read more information consistent with the vignettes about statin side effects.

*“it’s more, more sharings or, or… more people with credibility comes out with more findings to support this then, then I will stop it (statins), ya.” (P2, Vignette 1)*

## Discussion

Our findings highlighted factors patients perceived as important when evaluating OHI in the context of statin use in clinical practice. When encountering OHI that potentially affected statin use, patients considered the logical and neutrality of content and its source credibility, influenced by their prior knowledge and experiences. Patients would subsequently corroborate information from other sources to confirm the argument of the OHI.

When people have greater relevance and interest in the information, they think through the content of information critically [37]. Our research participants focused on the content of information when evaluating the vignettes, contrasting with other studies conducted among the public where research participants evaluated OHI based on heuristic cues such as website design, official touch and language [38]. This highlighted the importance of conducting health information research on people whose health decisions could be influenced by the information because they evaluated it differently from the public. Our study showed that patients mostly discerned the content of OHI based on their logical sense and own judgement with a lack of objective content credibility criteria. Tandoc et al. [39] described a similar evaluation process where people relied on their wisdom and instinct to authenticate news in social media.

Our findings illustrated the importance of content impartiality from the patients’ perspective. OHI with unbiased content and a neutral tone in the writing style appeared more trustworthy. These results were consistent with a systematic review showing that balanced views and transparency of information were the criteria people used to evaluate the quality of OHI [16]. Patients require balanced information to weigh the benefits and risks when evaluating conflicting information about their medication [40]. Both risks and benefits of health-related treatment are essential to be included when developing good-quality OHI. In our study, participants rejected OHI, which was intentionally misleading to arouse negative emotions. Similarly, Catellier et al. [41] noted that positive feelings towards health information were associated with trust, but negative affect did not. Our findings suggested that neutral and unbiased content are the keys to successful health communication to maintain patients’ trust.

Among various heuristics cues in information processing, our study showed that source credibility was the focus of patients when evaluating the vignettes. Evaluating the content credibility of messages is more difficult as a higher cognitive effort is required [39, 42]; therefore, patients looked at source credibility to guide them. Our participants knew how to look at the indicators for source credibility, such as credibility of authors, reputation of endorsing organisation and reference list. However, this does not translate to an accurate determination of information credibility and trust in our study. Patients were misled when similar source credibility indicators appeared in low-quality information. A similar phenomenon was described by Dissen et al. [43], where undergraduate students were unable to accurately determine the source credibility although they used factors such as website URL, domain suffix and website design to guide them. Source credibility remains an important criterion to use when evaluating OHI, especially in the era of information overloaded with a lack of time and effort to evaluate the content argument critically.

Our findings explained how patients’ existing beliefs and experiences influenced their trust in online information, supporting the theory of cognitive dissonance in the context of OHI-seeking behaviour [44]. According to the theory, individuals tend to maintain consistency with their cognitions, i.e. beliefs, values, opinions or attitudes [44]. In the context of information processing, our study participants trusted information consistent with their prior knowledge and experiences. This was observed in participants who trusted the poor-quality vignette when their negative beliefs about statins were consistent with the OHI. Belief-consistent information was perceived as more credible and convincing [45]. A psychological discomfort (cognitive dissonance) happens when an individual receives information that is inconsistent with their beliefs [44]. This discomfort motivates individuals to reduce the inconsistency by changing their beliefs or behaviours. In our study, participants experienced cognitive dissonance when they received new information from vignettes about statin side effects, which was inconsistent with their prior knowledge. They expressed that they would like to seek more information from different sources about statin side effects. This would potentially change their perception if other sources showed consistent content as the low-quality vignette. There is a need to guide people towards high-quality information because people might selectively seek, interpret or retain information that is consistent with statin misinformation [46].

### Implications for practice and research

A systematic review found that patient who sought OHI tended to have a better understanding of their diagnosis and motivated patients to ask questions during consultations; this would support patients making a shared decision with their doctors [7]. Providing good quality information is the initial and critical step in the shared decision-making process [47]. Doctors should provide patients with good quality OHI and guide them towards credible online sources to support patients with knowledge to make informed decisions. In addition, this study also provided an insight into how patients make decisions, including how poor-quality information might have negative cognitive and psychological influences on patients’ decision-making [48]. Keeping this in mind, doctors could adopt a more personalised approach in assessing their prior knowledge and beliefs about stain use, including OHI, before attempting to debunk online misinformation with patients.

The results of this study suggested several important aspects that should be included when designing a trustworthy OHI for patients. It is essential to focus on the content of OHI and have the input from the content and health communication experts in designing OHI. Local healthcare authorities and academic institutions should develop credible and updated OHI resources for patients and the public tailored to the local cultural background and context, serving as a primary source of reference for patients and the public. Our results informed the development of interventions to improve eHealth literacy, educating patients and the public about the tell-tale signs of a misleading OHI. We suggested educating patients and the public to rethink and challenge their perceptions and beliefs when appraising OHI and corroborating information from other sources. Also, official OHI developers should learn how to design websites and write content with a neutral stance and tone. A collaborative effort is needed between institutions, healthcare professionals and the public to use the Internet as a source of health information to improve healthcare.

Further research is needed to explore how people corroborate health information and to design interventions to help break the misinformation chain. Future research should explore how people with different eHealth literacy levels evaluate OHI, particularly those with low eHealth literacy. More research is required to develop effective interventions for improving eHealth literacy, especially in enhancing information appraisal skills.

### Strengths and limitations

The study was conducted among actual patients with health conditions who had relevant experience in making decisions about statins. Instead of retrospective recall about OHI that participants encountered previously, we used vignettes and think-aloud methods to allow real-time reflection of participants’ views. However, as the vignettes only captured a snapshot of a wide range of OHI about statins, the participants’ responses only reflected their views on the information contained in the vignettes. In this study, the researcher screened the eligibility criteria by asking the potential participants. The recruitment process could be improved by screening the participants using a simple self-administered questionnaire. As this study was conducted at a particular point in time, the trust in OHI over time could not be captured; this is important as trust in health information is a dynamic process which is intertwined with other factors and changes over time.

## Conclusion

Content quality, neutrality and source credibility were crucial factors when patients evaluated online information related to their health conditions. Our study illustrated the key ways patients trusted online information; when the information confirmed their prior beliefs and experience, and when they corroborated information with other sources.

### Supplementary Information

**Additional file 1: Appendix 1. **Vignettes. 

**Additional file 2: Appendix 2. **Interview guide and instruction script. 

## Data Availability

The data is available from the corresponding author on reasonable request.

## Abbreviations

OHI: Online health information
LMICs: Low-middle-income countries
