# Supplementary material for: How do patients with high cardiovascular risk evaluate online health information? A qualitative study
Source: BMC Prim Care. 2023 Nov 15;24:240. doi: 10.1186/s12875-023-02182-7 (PMC10647114; doi:10.1186/s12875-023-02182-7)
Supplement: Supplementary file 1 — Additional file 1: Appendix 1. Vignettes. [file 12875_2023_2182_MOESM1_ESM.pdf]

Appendix 1: Vignettes

Vignette 1 English: Statin danger

https://www.facebook.com/permalink.php?story_fbid=693199334075255&id=660236034038252&substory_index=0

Vignette 2 English: Statin aide effects: Weight the benefits and risks by Mayo Clinic. https://www.mayoclinic.org/diseases-conditions/high-blood-cholesterol/in-depth/statin-side-effects/art-20046013.

Vignette 1 BM: Bahaya Statin

https://www.facebook.com/permalink.php?story_fbid=693199334075255&id=660236034038252&substory_index=0

Vignette 2 BM: Faedah dan kesan sampingan ubat statin untuk warga emas

http://www.myhealth.gov.my/faedah-dan-kesan-sampingan-ubat-statin-untuk-warga-emas/ (This link is no longer accessible. Please email corresponding author to obtain the PDF format of this website).

Vignette 1 Chinese: http://www.360doc.com/content/16/0317/22/29110587_543177008.shtml

Vignette 2 Chinese: https://www.sinounitedhealth.com.cn/Html/Article/15055312880113.shtml

Selection of vignettes according to DISCERN tool

	DISCERN questions	V1 Eng	V2 Eng	V1 BM	V2 BM	V1 CN	V2 CN
1	Are the aims clear?	2	5	2	4	2	5
2	Does it achieve its aims?	3	5	3	5	3	5
3	Is it relevant?	4	5	4	5	4	5
4	Is it clear what sources of information were used to compile the publication (other than the author or producer)?	1	5	1	3	1	3
5	Is it clear when the information used or reported in the publication was produced?	5	5	5	5	5	5
6	Is it balances and unbiased?	1	5	1	5	1	5
7	Does it provide details of additional sources of support and information?	1	3	1	3	1	3
8	Does it refer to areas of uncertainty?	2	5	2	3	2	5
9	Does it describe how each treatment works?	2	5	2	4	2	4
10	Does it describe the benefits of each treatment?	1	5	1	5	1	4
11	Does it describe the risks of each treatment?	4	5	4	5	4	5
12	Does it describe what would happen if no treatment is used?	1	4	1	4	1	4

13	Does it describe how the treatment choices affect overall quality of life?	1	4	1	4	1	4
14	Is it clear that there may be more than one possible treatment choice?	3	4	3	4	2	4
15	Does it provide support for shared decision-making?	1	5	1	4	1	4
	Total score	32	71	32	63	31	65

Eng, English; BM, Bahasa Malaysia; CN, Chinese.

Rating scale: 1-5

Yes (5)- the quality criterion has been completely fulfilled

Partially (2-4)- The publication being considered meets the criterion in question to *some extent*. How high or low you rate ‘partially’will depend on your judgment of the extent of these shortcomings.

No (1) – the quality criterion has not been fulfilled at all.

Vignette 1 (English version)

2 11

INFO SIHAT

Community

Send Message

Home About Photos Videos More ▾ Like •••

INFO SIHAT

May 5, 2014 ·

STATIN DANGER

Statin drugs contribute to industry worth RM75 billion a year. Statin set by doctors to lower cholesterol rates in blood. It's not only the best selling medicine in its category, but it also provides a list of almost endless side effects that causes unfortunate consumers to suffer for a lifetime of high medical treatment costs. Statin is a dream come true of the Big Pharma industry.

What do you get with statin medicine
A study published in the American Journal of Cardiovascular Drugs found nearly 900 studies of bad effects associated with statin medicine. A recent Harvard study reveals a 50 percent increase in diabetes risk if you take statin medication. They call it increased risk of '' minor ". Researchers analyzed data over 2 million statin drug users have a higher level of liver failure, kidney failure, cataract and muscle weakness.

high blood pressure, stroke and peripheral neuropathy. More serious side effects found are as follows:

Myositis, muscle inflammation. The risk of muscle injury increases when some other medications are taken with statin use. For example, if you take statin and fibrate combinations - another drug reduces cholesterol - the risk of muscle damage increases compared to someone who takes statin only.

CPK level, or creatine kinase, muscle enzymes that when increased, can cause muscle pain, mild inflammation, and muscle weakness. This condition, although rare, can take a long time to recover.

Deadly Rhabdomyolysis (extreme muscle inflammation and damage)
With this condition, the whole body's muscles become sore and weak. A severely damaged muscle releases protein into the blood collecting in the kidneys. Kidney can be damaged trying to eliminate a large amount of muscle damage caused by statin use. This can ultimately lead to kidney failure or even death.

What are the real benefits of statin medicine?
Statin medicine is supposed to cure the cause of cardiovascular disease. However, the study analysis reported in the Journal of Negative Results in Bioperism last year concluded that despite the major increase in statin drug use, the number of people with heart attacks is not decreasing - but rather is increasing. Did you know that 50 percent of all heart attacks today are from people with normal cholesterol? Cholesterol is just a sign and not the cause of the problem here.

Statin medicine restricts the production of essential nutrients in your body, including CoQ10 essential enzymes, which are important for heart health and muscle function. The decreasing level of CoQ10 is common with statin drug use and it actually causes heart failure!

Statin medicine also damages cholesterol-based hormones. It causes thyroid dysfunction and decrease in all sex hormones. Low testosterone levels lead to low energy, sleepy, furious and depression. Researchers found a direct link between statin medicine and low testosterone after researching data in nearly 3.500 men who came to doctors complained about sexual problems.

There is now also a drug to lower cholesterol for people without cholesterol problems. Crestor has been approved for people with NO high cholesterol!

Peter H. Langsjoen, MD said, '' We are now in a position to witness the greatest medical tragedy of all time - never in medical history has been consciously created a life-threatening nutrient deficiency in millions of healthy people ".

Warning signs of statin

doctor immediately. Pregnant women or those with active or chronic liver disease can't use statin.

If you take statin drugs, tell your doctor about any medication from the counter or prescription, herbal supplements, and vitamins you are taking or planning to take them.

Statin drugs approved for use in the United States include:

Lipitor
Mevacor or Altocor
Zocor
Pravachol
Lescol
Crestor

Since being on the market, statin is among the most prescribed medications in the United States, with about 17 million users.

According to Dr. Mercola, statin is listed as one of the 11 types of drugs for dangerous cholesterol. In Malaysia, if you have been given a statin drug prescription by doctors, then be prepared for your final days.
The Secret Medicines

· See original · Rate this translation

Vignette 2 (English version)

Appointments at Mayo Clinic

Mayo Clinic offers appointments in Arizona, Florida and Minnesota and at Mayo Clinic Health System locations.

Request Appointment

Statin side effects: Weigh the benefits and risks

Print

Statin side effects: Weigh the benefits and risks

Statins are effective at lowering cholesterol and protecting against a heart attack and stroke, although they may lead to side effects for some people.

By Mayo Clinic Staff

Doctors often prescribe statins for people with high cholesterol to lower their total cholesterol and reduce their risk of a heart attack or stroke. While statins are highly effective and safe for most people, they have been linked to muscle pain, digestive problems and mental fuzziness in some people who take them and may rarely cause liver damage.

Statins include atorvastatin (Lipitor), fluvastatin (Lescol XL), lovastatin (Altoprev), pitavastatin (Livalo), pravastatin (Pravachol), rosuvastatin (Crestor, Ezallor) and simvastatin (Zocor, FloLipid).

Having too much cholesterol in your blood increases your risk of heart attacks and strokes. Statins block a substance your liver needs to make cholesterol. This causes your liver to remove cholesterol from your blood.

If you think you're experiencing side effects from statins, don't just stop taking the pills. Talk to your doctor to see if a change of dosage or even a different type of medication might be helpful.

What are statin side effects?

Muscle pain and damage

One of the most common complaints of people taking statins is muscle pain. You may feel this pain as a soreness, tiredness or weakness in your muscles. The pain can be a mild discomfort, or it can be severe enough to make your daily activities difficult.

However, researchers have found a "nocebo" effect when it comes to perceived muscle pain and statins. A "nocebo" effect means people who have negative expectations about a

Free E-newsletter

Subscribe to Housecall

Our general interest e-newsletter keeps you up to date on a wide variety of health topics.

Sign up now

Mayo Clinic does not endorse companies or products. Advertising revenue supports our not-for-profit mission.

Advertising & Sponsorship

Policy | Opportunities | Ad Choices

Mayo Clinic Marketplace

Check out these best-sellers and special offers on books and newsletters from Mayo Clinic.

FREE book offer — Mayo Clinic Health Letter

BRAND NEW - Back and Neck Health

Mayo Clinic on Digestive Health

Mayo Clinic Guide to Arthritis

Time running out - 40% off Online Mayo Clinic Diet ends soon

medication report experiencing the potential side effect at higher rates than the drug should cause.

The actual risk of developing muscle pain as a result of taking statins is about 5 percent or less compared with taking a pill that doesn't contain medication (placebo). However, studies have found that nearly 30 percent of people stopped taking the pills because of muscle aches even when they were taking a placebo.

A strong predictor you'll experience muscle aches when taking statins could be whether or not you read about the potential side effect.

Very rarely, statins can cause life-threatening muscle damage called rhabdomyolysis (rab-doe-my-OL-ih-sis). Rhabdomyolysis can cause severe muscle pain, liver damage, kidney failure and death. The risk of very serious side effects is extremely low, and calculated in a few cases per million people taking statins. Rhabdomyolysis can occur when you take statins in combination with certain drugs or if you take a high dose of statins.

Liver damage

Occasionally, statin use could cause an increase in the level of enzymes that signal liver inflammation. If the increase is only mild, you can continue to take the drug. Rarely, if the increase is severe, you may need to try a different statin.

Although liver problems are rare, your doctor may order a liver enzyme test before or shortly after you begin to take a statin. You wouldn't need any additional liver enzyme tests unless you begin to have signs or symptoms of trouble with your liver.

Contact your doctor immediately if you have unusual fatigue or weakness, loss of appetite, pain in your upper abdomen, dark-colored urine, or yellowing of your skin or eyes.

Increased blood sugar or type 2 diabetes

It's possible your blood sugar (blood glucose) level may increase when you take a statin, which may lead to developing type 2 diabetes. The risk is small but important enough that the Food and Drug Administration (FDA) has issued a warning on statin labels regarding blood glucose levels and diabetes.

The increase generally occurs when blood sugar levels are already higher than normal and fall in the prediabetes or diabetes range when you begin taking a statin.

Statins prevent heart attacks in people with diabetes, so the relevance of the mild increase in sugar values with statins observed in some people is unclear. The benefit of taking statins likely outweighs the small risk to have the blood sugar level go up. Talk to your doctor if you have concerns.

Neurological side effects

The FDA warns on statin labels that some people have developed memory loss or confusion while taking statins. These side effects reverse once you stop taking the medication. There is limited evidence to prove a cause-effect relationship, but talk to your doctor if you experience memory loss or confusion while taking statins.

There has also been evidence that statins may help with brain function — in people with dementia, for example. This is still being studied. Don't stop taking your statin medication before talking to your doctor.

Who's at risk of developing statin side effects?

Not everyone who takes a statin will have side effects, but some people may be at a greater risk than are others. Risk factors include:

- Taking multiple medications to lower your cholesterol
- Being female
- Having a smaller body frame
- Being age 80 or older
- Having kidney or liver disease
- Drinking too much alcohol
- Having certain conditions such as hypothyroidism or neuromuscular disorders including amyotrophic lateral sclerosis (ALS)

Drugs and food that interact with statins

Grapefruit juice contains a chemical that can interfere with the enzymes that break down (metabolize) the statins in your digestive system. While you won't need to eliminate grapefruit entirely from your diet, ask your doctor about how much grapefruit you can have.

Some drugs that may interact with statins and increase your risk of side effects include:

- Amiodarone (Cordarone, Pacerone), a medication for irregular heart rhythms
- Gemfibrozil (Lopid), another variety of cholesterol drug
- HIV treatments called protease inhibitors such as saquinavir (Invirase) and ritonavir (Norvir)
- Some antibiotic and antifungal medications, such as clarithromycin and itraconazole (Onmel, Sporanox)
- Some immunosuppressant medications, such as cyclosporine (Gengraf, Neoral, Sandimmune)

There are many drugs that may interact with statins, so be sure your doctor is aware of all the medicines you take when being prescribed with statins.

How to relieve statin side effects

To relieve side effects believed to be caused by statins, your doctor may recommend several options. Discuss these steps with your doctor before trying them:

- **Take a brief break from statin therapy.** Sometimes it's hard to tell whether the muscle aches or other problems you're having are statin side effects or just part of the aging process. Taking a break can help you determine whether your aches and pains are due to statins instead of something else.
- **Switch to another statin drug.** It's possible, although unlikely, that one particular statin may cause side effects for

you while another statin won't. It's thought that simvastatin (Zocor) may be more likely to cause muscle pain as a side effect than other statins when it's taken at high doses.
- **Change your dose.** Lowering your dose may reduce some of your side effects, but it may also reduce some of the cholesterol-lowering benefits your medication has. Another option is to take the medication every other day, especially if you take a statin that stays in the blood for several days. Talk to your doctor to determine if this is appropriate for you.
- **Take it easy when exercising.** Unaccustomed vigorous exercise might increase the risk of muscle injury. It's best to make changes in your exercise routine more gradually. Exercise causes muscle pain too, so it is sometimes difficult to know if the pain comes from the statin or the exercise in someone who just started an exercise program.
- **Consider other cholesterol-lowering medications.** Although statins are the most effective oral medications for lowering your cholesterol, other types of drugs also are available. Sometimes, taking a combination of cholesterol drugs can provide the same result with lower doses of statins.
- **Try coenzyme Q10 supplements.** Coenzyme Q10 supplements may help prevent statin side effects in some people, though more studies are needed to determine any benefits of taking it. Talk to your doctor first to make sure the supplement won't interact with any of your other medications.

Weigh the risks and benefits

Although side effects believed to be caused by statins can be annoying, consider the benefits of taking a statin before you decide to stop taking your medication. Remember that statin medications can reduce your risk of a heart attack or stroke, and the risk of life-threatening side effects from statins is very low.

If you have read about the potential side effects of statins, you may be more likely to blame your symptoms on the medication, whether or not they're truly caused by the drug.

Even if your side effects are frustrating, don't stop taking your statin medication for any period of time without talking to your doctor first. Your doctor may be able to come up with an alternative treatment plan that can help you lower your cholesterol without uncomfortable side effects.

Share Tweet

Jan. 14, 2020

Show references

See more In-depth

Products and Services

Newsletter: Mayo Clinic Health Letter — Digital Edition

Book: Mayo Clinic Family Health Book, 5th Edition

See also

After a flood, are food and medicines safe to use?

Arcus senilis: A sign of high cholesterol?

Birth control pill FAQ

Coenzyme Q10

Cholesterol concerns? Get moving

Cholesterol concerns? Lose excess pounds

Cholesterol level: Can it be too low?

Show more related content

Other Topics in Patient Care & Health Info

Diseases & Conditions A-Z

Symptoms A-Z

Tests & Procedures A-Z

Drugs & Supplements A-Z

Health Books

Healthy Living Program

Mayo Clinic Health Letter

Mayo Clinic Voice Apps

Any use of this site constitutes your agreement to the Terms and Conditions and Privacy Policy linked below.

Terms and Conditions
Privacy Policy
Notice of Privacy Practices
Notice of Nondiscrimination

Mayo Clinic is a nonprofit organization and proceeds from Web advertising help support our mission. Mayo Clinic does not endorse any of the third party products and services advertised.

Advertising and sponsorship policy
Advertising and sponsorship opportunities

A single copy of these materials may be reprinted for noncommercial personal use only. "Mayo," "Mayo Clinic," "MayoClinic.org," "Mayo Clinic Healthy Living," and the triple-shield Mayo Clinic logo are trademarks of Mayo Foundation for Medical Education and Research.

© 1998-2021 Mayo Foundation for Medical Education and Research (MFMER). All rights reserved.
