# Supplementary material for: How do patients with high cardiovascular risk evaluate online health information? A qualitative study
Source: BMC Prim Care. 2023 Nov 15;24:240. doi: 10.1186/s12875-023-02182-7 (PMC10647114; doi:10.1186/s12875-023-02182-7)
Supplement: Supplementary file 2 — Additional file 2: Appendix 2. Interview guide and instruction script. [file 12875_2023_2182_MOESM2_ESM.pdf]

Appendix 2: Interview guide and instruction script

I would like you to read some information on the internet.

- There are two pieces of information from two websites.
- The main purpose is for us to find out how you assess and react to these two pieces of information.
- This will help us to find out how patients interpret information from the internet.

Participant's task: When you read through the information, decide whether you would trust this information.

- I will ask you to think aloud as you read the information.
- Let me explain what I mean by "think aloud". It means I would like you to tell me everything you think as you work through each piece of information. Let me know every thought you have from the moment you read the information to the end after you finish reading the information.
- Please do not worry about planning how to say things or clarifying your thoughts.
- What I want is to hear your thoughts constantly.
- Sometimes you may need time to think quietly, if so, this is okay but please tell me what you thought through as soon as possible after you are finished.

Show patient Vignette V1 and Vignette V2 separately.

<u>**Retrospective interview session**</u>

The process of reading the online health information<br>• Can you tell me how you read through the information when you first received it from me?<br>▪ *[Prompt] What are the first things you look at?*<br>▪ *[Prompt] Which parts are important to you?*<br>▪ *[Prompt] Which part you ignored?*<br>•
Assess information and determine trust<br>• Would you trust the information?<br>*Score it on the Likert scale 1-5 (1 would not trust at all; 5 Mostly trusts)]*<br>*[prompt] Why do you trust/do not trust/unsure?*<br>• How do you evaluate this information?<br>• What makes you trust or don't trust online information?<br>*[Prompt]*<br>▪ ***Design*** *– Do you trust it based on the design and layout?*<br>▪ ***Language*** *– Would the language affect how you trust the information?*<br>▪ ***Author*** *– Would you look at who wrote the info? If YES, how it affects your trust?*<br>▪ ***Organizations*** *– Would the endorsed organization affect your trust?*<br>▪ ***Personalised information*** *– What do you think about people sharing their personal experiences? How you decide whether to trust the personal experiences shared on the website?*

- ***References*** *– Do you look at the references? What do you think about the references?*
- ***Sponsorship/advertisement*** *– For websites with advertisements, or sponsored by companies, does it affect your trust of the online health information?*

Any additional comments?

Thank the participant.
